# Editorial: Psychedelics as treatments for substance use disorders: exploring therapeutic potential, risks, underlying mechanisms of action, and implementation challenges

**DOI:** 10.3389/fpsyt.2023.1305478

**Published:** 2023-10-19

**Authors:** Brian S. Barnett, Anahita Bassir Nia, Nathan B. Sackett, Jeremy Weleff

**Affiliations:** ^1^Department of Psychiatry and Psychology, Center for Behavioral Health, Neurological Institute, Cleveland Clinic, Cleveland, OH, United States; ^2^Cleveland Clinic Lerner College of Medicine, Case Western Reserve University, Cleveland, OH, United States; ^3^Department of Psychiatry, Yale University School of Medicine, New Haven, CT, United States; ^4^Department of Psychiatry and Behavioral Sciences, University of Washington, Seattle, WA, United States; ^5^Center for Novel Therapeutics in Addiction Psychiatry, Department of Psychiatry and Behavioral Sciences, University of Washington, Seattle, WA, United States

**Keywords:** psychedelics, ketamine, psilocybin, addiction, substance use disorder

Though relatively under-researched compared to mood and anxiety disorders, the therapeutic applications of psychedelics for substance use disorders (SUDs) represent important possibilities for treating conditions that inflict tremendous morbidity and mortality. In recent decades, growing rates of mental illness (1) and social isolation (2) have intersected with increasingly efficient technologies in the processing, manufacturing, and dissemination of psychoactive substances to greatly exacerbate the prevalence and harms of SUDs. While psychotherapy, mutual support groups, and pharmacological interventions are effective for some with these conditions, for too many they prove inadequate means for ensuring long term recovery. For example, in an open label trial of sublingual buprenorphine vs. monthly-administered extended release naltrexone in opioid use disorder, at 24 weeks 57 and 65% of patients had relapsed, respectively (3). Other studies have identified a 40% attrition rate from 12-step recovery groups within the 1^st^ year of joining (4) and a 30% attrition rate for psychosocial SUD treatments in general (5). Numbers like these clearly indicate that novel approaches to treating SUDs are needed—could psychedelics help fill the gap?

In this Research Topic, we present seven articles exploring the therapeutic potential of classic and non-classic psychedelics for SUDs. Recognizing that psychedelic research has been prone to hype (6) and that unique questions related to potential therapeutic applications of psychedelics in patients with SUDs (such as psychedelics' addictive potential in this population) remain open, we sought to curate an article collection providing a balanced take on the current state of knowledge for this exciting, though sometimes controversial topic.

Zafar et al. present a wide-ranging review of observational studies and clinical trials investigating the therapeutic effects of classic and non-classic psychedelics in SUDs. They also extensively discuss different translational human neuropsychopharmacology techniques (such as functional magnetic resonance imaging and positron emission tomography) that should be employed in future work to enhance mechanistic understanding of how psychedelics might treat SUDs.

Whinkin et al. explore the treatment potential of ketamine for depression, anxiety, and psychosocial/spiritual distress in patients with SUDs, via a 2-year chart review on 18 patients with problematic substance use receiving ketamine-assisted psychotherapy. Statistically significant reductions in anxiety and depression rating scores were observed, suggesting further investigations in this area are warranted.

Goldfine et al. reviewed the human literature on the therapeutic potential of ketamine for alcohol use disorder (AUD), as well as alcohol withdrawal. Their findings regarding AUD included studies reporting reduced cravings, reduced alcohol consumption, and longer abstinence in patients receiving ketamine treatments, with longer periods of abstinence associated with receiving more psychotherapy sessions. The authors also identified literature demonstrating earlier resolution of alcohol withdrawal syndrome (AWS) and delirium tremens with the adjunctive use of ketamine in patients receiving benzodiazepine therapy during severe alcohol withdrawal.

Phan and Terry systematically reviewed the human literature on psychedelics, including ketamine, in the treatment of cannabis use disorder (CUD), a condition that has received relatively little attention in psychedelics research thus far. The authors identified one article assessing the open-label use of ketamine in conjunction with motivational enhancement therapy (MET) and mindfulness-based relapse prevention treatments for CUD. Patients in that study had a statistically significant reduction in cannabis use, though there were only eight participants. The authors also identified data from a placebo-controlled study of the serotonin 5-HT2c agonist lorcaserin in non-treatment seeking daily cannabis smokers indicating decreased cannabis use, though data were only reported for less than a week. Lorcaserin is not a psychedelic, though 5-HT2c agonism may play a role in psychedelics' potential anti-addictive properties, since this modulates dopaminergic activity in the ventral tegmental area-nucleus accumbens reward pathway (7).

Rounding out articles on human data, Brett et al. present a mini-review on psilocybin-assisted therapy for methamphetamine use disorder (MUD), a condition for which current pharmacological treatments offer little to no efficacy, and discuss the rationale for investigating psilocybin-assisted therapy for this indication. The authors note that since psilocybin-assisted therapy has demonstrated promising findings in clinical trials for AUD and tobacco use disorder, the possibility of transdiagnostic treatment potential justifies exploring its potential in MUD.

Zhornitsky et al. explore the potential of neuroplasticity as a therapeutic mechanism for psychedelics in patients with SUDs, as well as mental health conditions, by systematically reviewing the effects of psychedelics on markers associated with synaptic density. They found most eligible studies have investigated the non-classic psychedelic ketamine, with mixed findings for synaptic changes in the hippocampus and prefrontal cortex when ketamine is administered in single or repeated doses. Fewer studies have been conducted for other psychedelics, though these data indicate that markers associated with synaptic density can be enhanced under basal conditions and that stress-associated deficits can be reversed. Taken together, these findings suggest that psychedelics' abilities to normalize abnormal levels of synaptic markers in some brain regions could contribute to their treatment potential for SUDs.

Finally, in a compelling perspective piece, Black argues for an increased focus on the potential of psychedelic treatments for patients with SUDs. To do this demands that we work toward equitably employing psychedelic therapies, rather than maintaining the status quo in mental healthcare, which would likely see them primarily restricted to use in wellness markets or patients who are commercially insured. Among other points, Black observes that recruitment and training of diverse psychedelic clinicians, collaboration with mutual support groups, and a willingness to provide access to psychedelic treatments to higher-risk individuals in need of complex care will be essential to maximizing the potential societal benefits of psychedelic assisted therapy.

In summary, these articles provide a valuable view into the emerging field of psychedelics as potential treatment for SUDs. With growing interest among researchers in exploring this line of inquiry, we expect this Research Topic will prepare readers for deeper future engagement with an area of research that could yield important assistance in humanity's quest to ameliorate the widespread suffering wrought by SUDs.

## Author contributions

BB: Conceptualization, Writing—original draft. AB: Writing—review and editing. NS: Writing—review and editing. JW: Writing—review and editing.
